# THE ETHNIC-SPECIFIC EFFECTS OF BEHAVIORS ON INCIDENT GOUT WITHIN A LARGE MULTIETHNIC COHORT OF OLDER ADULTS

**DOI:** 10.1093/geroni/igz038.3157

**Published:** 2019-11-08

**Authors:** Mika D Thompson, Catherine M Pirkle, Yanyan Wu, Fadi Youkhana, Robert V Cooney, Lynne R Wilkens

**Affiliations:** 1 University of Hawai’i at Mānoa, Honolulu, Hawai’i, United States; 2 University of Hawai’i Cancer Center, Honolulu, Hawai’i, United States

## Abstract

Gout, a common rheumatic disease in older adults, disproportionately affects Pacific Islander and Black populations, relative to Whites; however, the ethnic-specific incidence and determinants remain severely understudied within these groups. We examined gout incidence and the effects of behavioral factors, including diet, physical activity, and smoking, on incident gout within a large multiethnic population of older adults from the Multiethnic Cohort Study linked to approximately 20 years of Medicare gout claims. Using samples of Black (N=15,660), Native Hawaiian (N=7,600), Japanese (N=32,923), Latino (N=21,793), and White (N=29,129) participants, we conducted multiple Cox regressions, producing hazard ratios (HR) and 95% confidence intervals (CI). Native Hawaiians had the highest incidence of gout (9.97 per 1,000 person-years), followed successively by Black, Japanese, White, and Latino participants. 3+ alcoholic drinks/day was associated with an increased risk, especially among Blacks (HR:1.55, 95%CI:1.23,1.94). Current smoking was associated with increased risk among Blacks (HR:1.28, 95%CI:1.10,1.49) and Japanese (HR:1.17, 95%CI:1.03,1.32). Better dietary quality tertiles were associated with a decreased gout risk for most ethnic groups, with the largest effect observed among Whites (HR[Q1vsQ3]:0.70, 95%CI:0.63,0.78), while vitamin C was weakly associated with a decreased risk of gout only among Native Hawaiians (HR:0.85, 95%CI:0.75,0.98) and Japanese (HR:0.91, 95%CI:0.85,0.98). Overall, notable ethnic differences were observed in both gout incidence and effects of modifiable behaviors. As the first study to examine the incidence and longitudinal determinants of gout utilizing several large samples of underrepresented ethnic groups, our findings offer crucial insights that may improve precision in preventing and treating gout.

